# Evaluation of Marginal Misfit of Metal Frameworks Welded by Gas-Torch, Laser, and Tungsten Inert Gas Methods

**DOI:** 10.1155/2018/9828929

**Published:** 2018-10-30

**Authors:** Wilson Matsumoto, Paula Pastana Beraldo, Rossana Pereira de Almeida, Ana Paula Macedo, Beatriz Roque Kubata, Takami Hirono Hotta

**Affiliations:** ^1^Department of Dental Materials and Prosthodontics, Dental School of Ribeirão Preto, University of São Paulo, Ribeirão Preto 14040-904, Brazil; ^2^Private Practice, Ribeirão Preto 14025-010, Brazil

## Abstract

**Purpose:**

The objective of this study was to evaluate the marginal fit and the flexural resistance of nickel-chromium frameworks welded by different techniques, gas-torch, laser, and tungsten inert gas (TIG), compared with that of frameworks made via one-piece casting.

**Methods:**

To evaluate the marginal fit, a master model was fabricated simulating four implants. Transfers and replicas were used to transfer the positions of the implants to the model, using a silicone matrix. The bars were waxed up and casted. Three assessments of misfit were performed for each implant using a stereomicroscope before and after welding in two predetermined regions, totaling six readings for each implant. To evaluate the flexural resistance, one group was made casting the specimens in one piece. Other 3 groups using gas-torch, laser, and TIG welding techniques were made after sectioned transversally. The data showed normal distribution and two-way ANOVA for marginal fit and one-way ANOVA for flexural resistance, and Tukey's posttest (*α*=0.05) was performed.

**Results:**

For the marginal fit, the three welding methods presented similar results and were different from one-piece casting. For the flexural resistance, significant differences were observed among the studied groups (*p* < 0.001), and the one-piece group presented higher resistance compared to the three welding techniques.

**Conclusions:**

Within the limits of this study, the three welding techniques yielded similar misfit results, and the laser and TIG techniques presented similar flexural resistance but lower than gas-torch and one-piece casting.

## 1. Introduction

The success of an oral rehabilitation procedure is associated with the use of techniques and materials that enable a fixed prosthesis supported by teeth or implant a uniform distribution of forces and passive fit [1]. Achieving good adaptation with regard to prosthetic restorations is difficult, especially for extensive or one-piece cast framework [2, 3]. This fact is considered a common cause of fixed prosthesis failure due to alloy shrinkage after solidification or a lack of parallelism between the retainers. In this situation, welding is a method that can provide better adaptation than that achieved via one-piece metal structure [2, 4, 5].

Major advantages associated with welding include the option of using framework segments reducing the likelihood of failures occurring during manufacture, improving the adaptation and distribution of the masticatory forces, and producing structures with lower distortion and better adaptation [5]. A passive fit is essential for maintaining mechanical and biological balance that decreases the loading on the abutments [6]. Engineering advances have contributed to the development of new welding techniques and equipment as an alternative to conventional gas-torch techniques. These new modalities include laser [7] and tungsten inert gas (TIG) welding [8]. These different welding methods have associated advantages and disadvantages. The gas-torch technique causes oxidation, porosity, and overheating that can cause distortion [9] and is contraindicated for cantilever and extensive prostheses. Laser welding is advantageous because it entails the application of concentrated energy in a short time and less distortion; however, it is reportedly associated with variable strength results [10], unlike TIG welding which yields values similar to one-piece casting, with less porosity, and is indicated for sites with high resistance requirements [11].

Given the above-described considerations, investigations aimed at identifying welding processes that result in high-quality prostheses with suitable properties, or at least properties that are within clinically acceptable limits are of fundamental importance. The objective of the current study was to evaluate the marginal misfit and the flexural resistance of different methods of soldering parameters associated with nickel-chromium (Ni-Cr) frameworks welded by gas-torch, laser, and TIG techniques. The null hypothesis was that the welding method has no influence on the vertical misfit and the flexural resistance of Ni-Cr frameworks.

## 2. Materials and Methods

### 2.1. Master Model Fabrication

The master model was constructed from a transparent acrylic resin lower jaw model (Nacional Ossos, Jau, SP, Brazil) with four replicas of mini conical abutment (4.1 mm in diameter; Neodent, Curitiba, PR, Brazil), simulating a fixed prosthesis Branemark-type protocol with 4 implants.

### 2.2. Stone Cast Fabrication

To transfer the position of the mini conical abutment replicas, four square transfers (Neodent, Curitiba, PR, Brazil) were adapted and joined together with dental floss and acrylic resin (Duralay, Reliance, Worth Illinois, USA). After polymerization, the resin and dental floss set were cut between transfers with a #170L carbide bur to compensate for acrylic resin polymerization shrinkage. The sectioned parts were then rejoined with resin (Duralay, Reliance, Worth, Illinois, USA). After acrylic resin polymerization, the splinted transfers were removed from the master model.

The replicas were adapted to the splinted transfers, and the assembly was placed in a silicone matrix (Zetaplus, Dentsply, Petrópolis, RJ, Brazil) that was filled with type IV stone (Durone, Dentsply, Petrópolis, Brazil) and allowed to set on the workbench for 60 min. The model was then removed from the silicone matrix, and splinted transfers were removed from the stone cast and readapted on the master model to verify fit accuracy. The process was repeated to make 30 stone casts, which were subjected to different welding methods: gas-torch (*n*=10), laser (*n*=10), and TIG (*n*=10).

### 2.3. Wax Pattern Fabrication

Mini conical plastic cylinders (Neodent, Curitiba, PR, Brazil) were adapted onto the models, and wax-ups of the frameworks were made. Twenty wax patterns were cut in the middle of the distance between the plastic cylinders to derive the laser and TIG groups, with a 0.1 mm thick chrome-platinum blade.

### 2.4. Metal Structure Casting

The wax patterns were invested with high-fusing phosphate bonded investment (Heat Shock, Polidental, Cotia, SP, Brazil) in accordance with the manufacturer's instructions. Samples were cast with a centrifugal machine equipped with a gas oxygen torch (EDG, Sao Carlos, SP, Brazil), using a Ni-Cr alloy (Fit Cast-SB Plus, Talmax, Curitiba, PR, Brazil). Casted samples were finished with airborne-particle abrasion with 100 *μ*m alumina at 90 lib/pol^2^. Ten one-piece cast frameworks were adapted in the master model to perform gap measurements between the metal framework and mini conical replica.

### 2.5. Measurement of One-Piece Cast Framework Discrepancies

Measurements (60x, scale 7.69 *μ*m) were performed using an S8AP0 stereomicroscope (Leica, Leica Microsystems, Wetzlar, Germany) and Leica Application Suite V3 software (Leica Microsystems).

### 2.6. Gas-Torch Welding Group

After measurement, samples were cut between the retainers with a 1 mm thick carborundum disk, constituting the welding gas-torch group (*n*=10). Samples were indexed with acrylic resin (Duralay, Reliance, Worth, Illinois, USA) and invested with phosphate investment (Heat Shock, Polidental, Cotia, SP, Brazil). Gas-torch welding was conducted in accordance with the manufacturer's instructions (Dentorium, Labordental, Sao Paulo, SP, Brazil), and the samples were bench cooled at room temperature. The models with the sectioned metal frameworks were randomly divided into laser welding technique (*n*=10) and TIG welding technique (*n*=10) groups.

### 2.7. Laser Welding Group

A desktop laser welder (Dentaurum, Ispringen, Germany) was used to perform the laser welding technique. It was programmed at 310 V, 9.0 ms, and a pulse frequency of 4 focus, so that the welding reached deeper, and 4 welding points were made at anterior, posterior, superior, and inferior areas. Subsequently, the machine configuration was changed to 270 V, 9.0 ms pulse, and 8 focus to facilitate a wider and shallower area of welding to completely fill the sectioned line of the samples.

### 2.8. TIG Welding Group

A welding machine (NTY 60 model, Kernit, Indaiatuba, SP, Brazil) set to 60 A and a time of 120 ms was used to perform TIG welding. The machine was fitted with a tungsten electrode centered within the ceramic nozzle, following the manufacturer's recommendations. The time for preflow and postflow of argon gas was 2.0 s. Subsequently, the metal frameworks were transferred from the stone cast to the master model to analyze the interface between the frameworks and the mini conical replicas. Frameworks were screwed with a torque of 10 N·cm applied via a manual torque wrench (Neodent).

### 2.9. Measurement of Metal Framework Discrepancies

All welded groups were analyzed with a stereomicroscope under the same conditions applied to the one-piece cast group. The same operator performed three readings at two different positions (buccal and lingual) for each implant, totaling 6 measurements, and the mean was calculated for each implant. All groups were measured before and after welding.

### 2.10. Flexural Resistance Test

The cylindrical specimens, 3 mm in diameter and 40 mm in length, were cast in Ni-Cr alloy (Fit Cast-SB Plus, Talmax, Curitiba, PR, Brazil) using one-piece technique. Ten specimens were used as control group or one-piece group, and the other specimens were sectioned in two parts at middle distance and grouped according to the welding techniques in gas-torch, laser, and TIG groups.

The specimens were submitted to the three-point flexural test in the universal testing machine (EMIC DL 2000, São José dos Pinhais, PR, Brazil), where a 500 kg load cell was attached at a speed of 0.5 mm/min. Previously, markings were made that identified the center of the specimen, the weld location, and the location where the test piece should rest. The force, carried by a steel tip of 3.0 mm in diameter, was applied to the welded site until the fracture of the specimen had occurred. The forces necessary to fracture the bars were recorded.

### 2.11. Statistical Analysis

Statistical analysis was performed with SPSS for windows (SPSS 17.0 statistics software, SPSS Inc., USA), and *p* values of less than 0.05 were considered significant. A misfit comparison was done using the two-way analysis of variance (ANOVA). A flexural resistance comparison was done using the one-way ANOVA. Tukey's test and Student's *t*-test were performed to compare the groups.

## 3. Results

Table 1 shows the mean and standard deviation of the vertical misfit of the one-piece casting framework and different techniques before and after welding. Statistical analysis confirmed a normal distribution. In the two-way ANOVA, there was a statistically significant difference between welding types (*p*=0.004) and no significant difference for implant position (*p*=0.054). There was no statistically significant difference for the interaction of variables (*p*=0.153).

Tukey's posttest was performed to identify differences between welding types. Tukey's test showed similarity between the experimental groups before welding, which differed from the one-piece group. Tukey's test showed that the gas-torch, laser, and TIG groups were similar.

Student's *t*-test for paired samples was used to compare the results obtained before and after welding, and the gas-torch technique yielded results that were statistically similar before and after welding (*p*=0.56). This was not the case for the laser group (*p*=0.009) or the TIG (*p*=0.002) group, in which there was higher marginal misfit after welding.

Table 2 shows the mean and standard deviation of the flexural resistance (MPa) associated with different types of welding.

For flexural resistance, a significant difference was observed between the groups (*p* < 0.001). After Tukey's test, it was verified that the one-piece group presented greater resistance when compared to the welded groups (*p* < 0.001). Gas-torch group presented higher resistance than the laser (*p*=0.049) and TIG (*p*=0.026) groups. And there was no significant difference between the laser and TIG groups (*p*=0.994).

## 4. Discussion

The use of welding is justified by the advantage of working with segments of the prosthesis, reducing possible failures during the design of the metal structure and allowing better adaptation of the framework, uniform distribution of forces and, consequently, minimization of traumas and failures in the prosthesis. However, as several problems related to the welding process have been reported in the literature, considering the type of alloy [5, 9, 11], oxidation of the faces to be joined by the weld, porosity in the joint, and overheating of the bonding site during the welding process [2], welding can still be considered a process that demands more studies and research with materials, techniques, and equipment. In this study, the null hypothesis was partially accepted because the results obtained for the marginal misfit from the gas-torch, laser, and TIG groups did not differ significantly; however, the flexural strength was higher for the gas-torch.

The growth in the use of base alloys and the fact that prosthesis obtained by one-piece casting does not offer desirable adaptation, there is still a concern in welding metal segments, using techniques that do not produce fragile points and be practically and commercially viable. In addition, prosthesis must have the necessary characteristics to resist the chewing force and the high loads coming from bite forces especially the force in the occlusogingival direction.

Despite the impossibility of perfect adaptation between the tooth and metal framework due to various clinical and laboratory procedures [1, 2, 12, 13], the marginal fit of a prosthetic restoration is fundamental for its clinical success in the long term [14], and the presence of microgaps can affect the mechanical performance of the prosthesis [15]. This fact has prompted the elaboration of many studies with the objective to minimize problems associated with marginal discrepancy [14, 16]. In implant-supported prostheses, the lack of passivity of the metal framework on abutments can cause biological complications and prosthetic component failures [13]. Fixed prostheses should have a passive fit with osseointegrated implants because it is very difficult to predict what will happen clinically when a given degree of misfit is present [17]. A prosthesis misfit can cause overload on the adjacent bone and on prosthetic components, resulting in fatigue, screw loosening or fracture, and abutment screw fracture. This may compromise the integrity of the implant bone interface [18]. What does or does not constitute an “accurate” fit with regard to clinical interface relationships has been shown to be difficult to define [19].

Technical improvements have been made in the field of implants to enhance the fit accuracy of the abutment/prosthesis interface [16]. The current study examined the marginal misfit of the Ni-Cr frameworks after different welding procedures, gas-torch (brazing), laser, and TIG methods, and compared the results to those derived from a one-piece cast group. Laser welding has favorable features including heat reduction in the worked area [11] and elimination of the use of solder alloys and investments. The welding process can be performed directly in the plaster model [20] and even directly in the oral cavity [21] resulting in reduced dimensional changes and working times. TIG welding is conducted in an argon gas environment and can join parent metals with or without filling solder. Therefore, corrosion resistance can be increased due to the absence of galvanic effects at the joint. TIG welding is not commonly used in dentistry, but some studies have demonstrated the superiority of the technique over brazing methods in cobalt-chromium and Ni-Cr-based alloys, with similar results to those of laser welding [8, 10, 12]. TIG involves a very rapid cooling process, and all procedures can be performed directly over the definitive cast. The union is obtained via the use of an electric arc with a continuous current within an inert atmosphere (argon) [20].

The highest values of misfit were found in the one-piece group (31.2 ± 12.9 *µ*m) (Table 1), however, within the values considered clinically acceptable and below the average values found in other studies [12, 22–24]. One-piece prosthesis casting is a very sensitive technique, which requires an optimal working protocol and a skilled technician.

Before welding, the experimental groups exhibited similar marginal misfit and the one-piece control group exhibited a statistically significant difference, with average values of major discrepancy, confirming a greater possibility of alterations [13]. After welding, the gas-torch, laser, and TIG groups remained statistically similar to each other, but the gas-torch group differed significantly from the one-piece group. In the current study, after welding, the TIG and laser groups yielded results that did not differ significantly from those of the one-piece group, which was unexpected because it was predicted that the welding process would minimize the negative effects of one-piece casting [13]. These results are concordant with those reported by de Castro et al. [25], who concluded that there was no significant difference between one-piece cast, laser, and TIG welding frameworks.

The results of the current study differ from those of other studies [26–28], in which the authors affirmed that base metal castings do not provide a satisfactory level of fit unless additional refinement treatment is performed. Another study by Barbi [16] showed that TIG welding produced better results than the brazing or laser method. The difference between the one-piece and gas-torch groups in the current study may be related to the need for greater skill and experience of the professional when utilizing the gas-torch welding technique, as it requires standardization and control to achieve consistent and reliable results [2, 4]. Misfits are always present, regardless of the joining procedures used [22].

The gas-torch group presented specimens with excellent resistance values, conferring to this method validity for its use by the laboratories, as an efficient practice. However, the results showed a high standard deviation, indicating that it is a very sensitive technique. This technique is considered as difficult to execute, requiring a skilled professional [2, 9]. Despite the deficiencies, the gas-torch technique continues to be one of the most used by dental laboratories, mainly due to the low cost.

When the equipment is correctly calibrated, the laser and TIG techniques does not require much skill by the operator, compared to gas-torch technique, minimizing the occurrence of unsatisfactory results, as observed by the lower standard deviation of these techniques. Although previous studies, with the TIG technique, were performed with engineering-adapted machines, the results were similar or even superior when compared with other methods [11, 20, 29, 30].

The gas-torch technique produces a large area of the heat-affected zone (HAZ), around 2 mm, whereas the laser energy produces only 0.5 mm of a highly concentrated, localized, and rapid heat source (milliseconds) [7]. The welding performed in the TIG groups presented the same characteristics as the laser technique. This explains the fact that the specimens of the gas-torch group with greater strength always fractured in the region of the heat-affected zone, with complete separation of the two fractured halves and the lower resistance specimens fractured in the welded sites due to the porosities. The TIG and laser groups had their fractures always in the weld sites, but without the complete separation of the halves, showing ductility of the metal provided by minimal structural change and also by the absence of a soldering alloy. The great challenge, in the welding process, is the search for frameworks with the same structural characteristics as the frameworks obtained by one-piece casting with minimal structural changes. The greater the heat-affected zone, the greater the risk of distortions. One can consider that according to these parameters, laser and TIG techniques are more suitable than gas-torch. Since the gas-torch technique uses a third material to effect the welding, which does not occur in the other two techniques, there is improvement in the characteristics of the welded area, both in relation to the strength and to the adhesion of the ceramic to the metal structure.

The results of the misfit and the flexural resistance of this study show that the TIG and laser welding techniques produced good results, and these techniques could be considered as viable as they are easy to perform. The three weld techniques used in this study demonstrated to be able to improve the clinical conditions of a prosthesis by lowering the misfit and producing adequate flexural resistance [31] in spite of lower than the one-piece casting.

One limitation of this study was that the measurements of the misfit were performed in a single plane, not three dimensionally. Future studies should incorporate three-dimensional marginal misfit evaluation.

## 5. Conclusions

Within the limits of this study, after welding the three techniques, it yielded similar misfit results, and the laser and TIG welding techniques presented similar flexural resistance but lower than the gas-torch and the one-piece casting. Misfits currently remain inevitable regardless of the joining procedures used.

## Figures and Tables

**Table 1:** Mean and standard deviation of the marginal discrepancy (*μ*m) associated with the different techniques before and after welding.

	Type of welding	Mean (*μ*m)	Standard deviation (*μ*m)	*N*
Before	One-piece	31.2	12.9	10
	Torch	19.2	7.0	10
	Laser	20.7	2.2	10
	TIG	21.3	3.1	10

After	One-piece	31.2	12.9	10
	Torch	22.3	9.1	10
	Laser	23.7	3.8	10
	TIG	23.8	3.9	10

**Table 2:** Mean and standard deviation of the flexural resistance (MPa) associated with different types of welding.

Type of welding	Mean (MPa)	Standard deviation (MPa)	*N*
One-piece	1874.7	212.6	10
Torch	1313.5	394.2	10
Laser	987.8	158.8	10
TIG	956.8	253.3	10

## Data Availability

The PDF data used to support the findings of this study are available from the corresponding author upon request.
